# Integration of hyperspectral imaging and transcriptomics from individual cells with SpectralSeq

**DOI:** 10.1101/gr.280014.124

**Published:** 2025-08

**Authors:** Yike Xie, Abbas Habibalahi, Ayad G. Anwer, Kanu Wahi, Jacqueline Bailey, Francis Lin, Catherine Gatt, Emma M.V. Johansson, Tatyana Chtanova, Jeff Holst, Ewa Goldys, Fabio Zanini

**Affiliations:** 1 School of Clinical Medicine, UNSW Sydney, Sydney, New South Wales 2052, Australia;; 2 School of Biomedical Engineering, UNSW Sydney, Sydney, New South Wales 2033, Australia;; 3 School of Biomedical Sciences, UNSW Sydney, Sydney, New South Wales 2052, Australia;; 4 School of Biotechnology and Biomolecular Sciences, Faculty of Science, UNSW Sydney, Sydney, New South Wales 2052, Australia;; 5 Flow Cytometry Unit, Mark Wainwright Analytical Centre, UNSW Sydney, Sydney, New South Wales 2033, Australia;; 6 UNSW Cellular Genomics Futures Institute, UNSW Sydney, Sydney, New South Wales 2052, Australia;; 7 Evolution and Ecology Research Centre, School of Biological, Earth and Environmental Sciences, UNSW Sydney, Sydney, New South Wales 2033, Australia

## Abstract

Microscopy and omics are complementary approaches to probe cellular molecular states in health and disease, combining granularity with scalability. However, integrating both imaging- and sequencing-based assays on the same cell has proven challenging. This study demonstrates a new approach called SpectralSeq that combines hyperspectral autofluorescence imaging with transcriptomics on the same cell. SpectralSeq is applied to Michigan Cancer Foundation-7 (MCF-7) breast cancer cells and identifies a subpopulation of cells exhibiting bright autofluorescence rings at the plasma membrane in optical channel 13 (λ_ex_ = 431 nm, λ_em_ = 594 nm). Correlating the presence of a ring with the gene expression in the same cell indicates that ringed cells show higher expression of apoptosis-related genes and lower expression of ATP production genes. Furthermore, correlation of cell morphology with gene expression reveals downregulation of multiple spliceosome members in larger MCF-7 cells. Multiple genes exhibit consistent expression across cell sizes but varied exon usage. Finally, correlation between gene expression and fluorescence within the spectral range of nicotinamide adenine dinucleotide hydrogen (NADH) provides insights into the metabolic states of MCF-7 cells. Overall, SpectralSeq links optical spectrum with internal molecular states, offering a single streamlined workflow for single-cell resolution studies integrating spectral, morphological, and transcriptomic analyses.

Single-cell omics are accelerating our understanding of cell biology (Vandereyken et al. 2023), revealing new cell types and states in culture as well as in vivo across tissues, organs, and organisms (Camp et al. 2017; Liu et al. 2022; Qiu et al. 2022; Zanini et al. 2024). Major breakthroughs have increased the throughput of scRNA-seq and related technologies to analyze thousands of cells (Klein et al. 2015; Rosenberg et al. 2018; Aicher et al. 2019; Bageritz and Raddi 2019), and even millions in some cases (Zheng et al. 2017; Rosenberg et al. 2018), as well as integrating distinct omic modalities into single experiments (e.g., CITE-Seq [Stoeckius et al. 2017], scATAC+scRNA-seq [Zhang et al. 2022]). However, progress in combining omics with additional, complementary measurements from the same cell remains limited (Sarma et al. 2019; Camunas-Soler et al. 2020; Chen et al. 2022; Camunas-Soler 2024). Multimodal characterization of individual cells would benefit biomedical applications including cancer and infectious disease, given that both malignant and virus-infected cells can be genetically (Kleensang et al. 2016), transcriptionally (Zanini et al. 2018), and metabolically (Guillaume-Gentil et al. 2017) diverse within a single culture.

Fluorescence-based microscopy is used to explore cellular heterogeneity for decades. Hyperspectral imaging, which uses narrow band filters on both excitation and emission frequencies to detect the autofluorescence of intrinsic biomolecules, has emerged in recent years as a particularly information-rich methodology to study cytometabolic heterogeneity (Gosnell et al. 2016b; Campbell et al. 2019; Bertoldo et al. 2020). This technique can assign each cell multiple features related to morphology, spectral profile, and combinations of the two (e.g., spectral features with a specific subcellular localization). Hyperspectral data is used to train machine learning models aimed at identifying biomedically relevant phenotypes, including developing signatures of melanoma (Gosnell et al. 2016b; Knab et al. 2024), diabetes (Gosnell et al. 2016b), and other diseases (Gosnell et al. 2016a). In addition, hyperspectral features are linked with molecularly defined and interpretable cellular states, including cell types (Gosnell et al. 2016b) and cell cycle (Campbell et al. 2019). These features make it possible to develop noninvasive diagnostics relevant for biology and medicine, particularly important in areas such as reproductive medicine, for genetic (aneuploidy) diagnostics of early embryos (Tan et al. 2022), mitochondrial disease (Gosnell et al. 2016a), and other conditions (Campbell et al. 2023a,b).

Multiple groups have attempted to combine optical and sequencing methods at the single-cell level into a single experimental approach (Lamanna et al. 2020; Wehling et al. 2022). Spatial transcriptomic technologies have advanced tremendously, with commercial solutions like Visium and Xenium (10x Genomics), Slide-Seq (Rodriques et al. 2019), and STOmics (BGI). These methods capture large areas of ex vivo tissues and focus on (i) each cell's location relative to others, and (ii) the location of each detected RNA molecule relative to its cell of origin (e.g., MERFISH [Xia et al. 2019]). These technologies incur significant tradeoffs either on spatial resolution or on molecular sensitivity.

Techniques to achieve both high transcriptome quality and high spatial resolution have been proposed. Live-seq uses an atomic force microscope with a hollow cantilever tip to extract only a fraction of the cytoplasmic RNA from a living cell (Chen et al. 2022). Its platform, FluidFM (Dörig et al. 2010), requires a high level of expertise and currently offers limited throughput in terms of cell numbers. Laser dissection and capture suffer from similar limitations in terms of throughput and user skill requirements (Civita et al. 2019; Ong et al. 2020). Microfluidic droplet generators such as Drop-Seq (Bageritz and Raddi 2019), inDrop (Zilionis et al. 2017), and commercial solutions (e.g., 10x Genomics, BD Rhapsody) can be coupled to a microscope (Gérard et al. 2020); however, the droplets are not addressable, therefore the link between cell image and transcriptome is lost. Fluidigm C1 microfluidic chips (DeLaughter 2018) and the Takara ICELL8 (Goldstein et al. 2017) system can be used in theory to relate optical and transcriptomic features from the same cell. However, both technologies have limited image quality and are used primarily to verify cell capture.

Therefore, we aim to develop a novel approach with lower technical requirements, better image quality, and higher throughput than other methods. Here, we report SpectralSeq, a novel approach that combines hyperspectral autofluorescence imaging with transcriptomics from the same cell and scales to hundreds of cells in a single experiment. To demonstrate the utility of SpectralSeq, we used Michigan Cancer Foundation 7 (MCF-7) cells, a widely studied human breast cancer cell line (Lee et al. 2015), and correlated both spectral and morphological features of each cell with gene expression and preferential exon usage.

## Results

### Designing a workflow for single-cell imaging and transcriptomics

**Figure 1.**
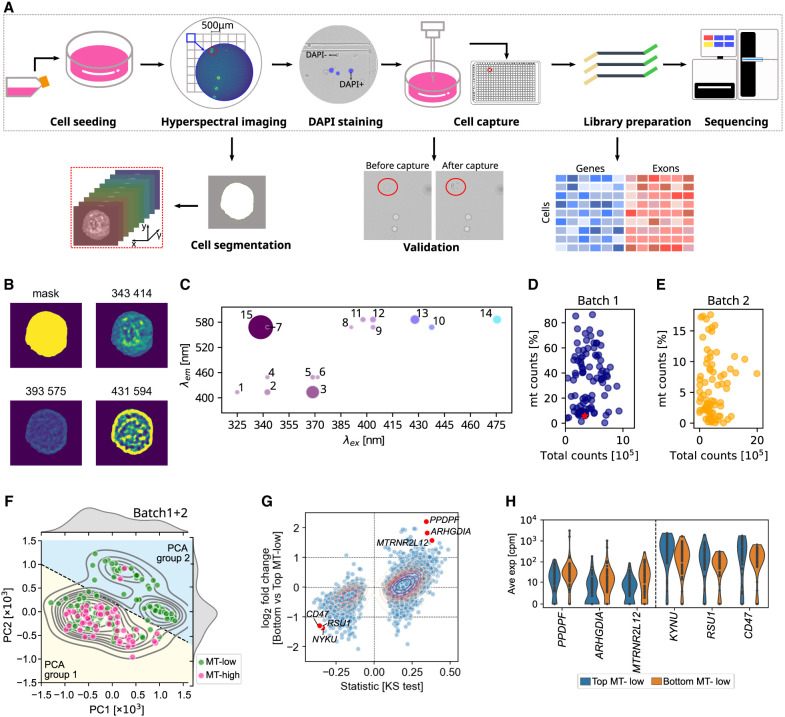
SpectralSeq integrates hyperspectral imaging and transcriptomics from the same cell. (*A*) Schematic of SpectralSeq's experimental and computational workflow. (*B*) Segmentation mask and select autofluorescence channels for a representative cell. (*C*) Autofluorescence spectrum for the same cell as in *B*. Each dot indicates a channel with the channel number labeled (Supplemental Table S1). Dot size indicates fluorescence intensity detected in each channel and dot color is a left-right gradient corresponding to excitation wavelength. All pixels within the segmentation mask were averaged. (*D*) Total reads per cell versus percentage of mitochondrial over total reads for experimental batch 1, with the same cell as in *B* highlighted as a red star. (*E*) As in *D* but for experimental batch 2. (*F*) Hyperspectral PCA for both batches combined, highlighting two groups of cells: PCA group 1 (yellow background) and PCA group 2 (blue background). (*G*) Scatterplot of the log_2_ fold change between MT-low cells in PCA groups 1 and 2 (*y*-axis) and signed statistics from KS test (*x*-axis). Genes with median number in either subgroup as 0 were filtered out. Some genes that are lower or higher expressed in bottom MT-low cells are labeled. (*H*) Violin plot showing the expression of highlighted genes in *G* in top and bottom MT-low cells.

To characterize both optical and transcriptomic features within the same cell, we developed SpectralSeq, a workflow integrating microscopy and scRNA-seq (Fig. 1A; see Methods for a detailed protocol). Briefly, breast cancer MCF-7 cells (Comşa et al. 2015) were seeded onto a gridded cell culture dish at low density and imaged on an Olympus IX83 inverted microscope customized for collection of brightfield and 15-color hyperspectral autofluorescence (Supplemental Table S1; see Fig. 1A for a representative false-color image of channel 2). After imaging, each cell culture was transferred to a different laboratory, where individual cells were isolated using an automated cell picker and deposited into a 384-well PCR plate. To ensure the capture of viable cells without compromising the hyperspectral images, cells were stained with DAPI, a marker of cell membrane breaching and death, after imaging and before capture. Low-resolution brightfield images before and after capture were used to verify successful cell isolation (Fig. 1A). Smart-seq2 was scaled down for cost-effective transcriptome library preparation (Zanini et al. 2018). To optimize single-cell capture within 1 h after hyperspectral imaging, we evaluated cell density for minimizing simultaneous capture of surrounding cells (Challenge 1) and trypsin dilution for cell detachment (Challenge 2). A density of 5000 cells/dish and a 1:10 trypsin dilution lifting cells gently within 30 min without disturbing surrounding cells ensured single-cell capture efficiency (Supplemental Table S2).

A pilot experiment using 30 cells with and without imaging steps indicated that imaging and picking had a negligible impact on RNA quality as evaluated by mapped read counts (Supplemental Fig. S1A) and the fraction of mitochondrial to total reads (Supplemental Fig. S1B). Gene expression in nonimaged control cells was highly correlated with SpectralSeq-processed cells (Spearman's ρ = 0.83, *P*-value below machine precision) (Supplemental Fig. S1C), confirming that SpectralSeq itself does not cause major changes in gene expression.

### SpectralSeq integrates optical and molecular information at the single-cell level

Given the promising outcome of the initial feasibility study, two larger batches were collected. Batch 1 (126 MCF-7 cells) was collected without DAPI staining, whereas batch 2 (83 MCF-7 cells) included the DAPI staining step. To quantify the hyperspectral images, a semiautomated cell segmentation pipeline using fake colors was developed (see Methods; Supplemental Fig. S1D). The segmentation mask was confirmed visually and was used to produce images with diverse patterns at subcellular resolution (Fig. 1B). To quantify the spectral information of each cell, the optical intensity from all pixels within the segmentation mask was averaged and combined into a cell-specific spectral fingerprint, or cell spectrum (Fig. 1C).

Quality controls were implemented to verify the efficiency of SpectralSeq, including visual confirmation of a single cell in the brightfield imaging channel (see Methods). In batch 1, out of 126 cells, three lacked good hyperspectral data, and of the remaining 30 suspicious doublets, 27 were confirmed as doublets upon further visual inspection using before-and-after capture images from CellCelector. The doublets were intentionally selected due to their close association, resembling sister cells from the division of a mother cell (Supplemental Fig. S2; Supplemental File S1). Differential gene analysis between these doublets and single cells showed that the doublets exhibited higher expression of genes associated with cell cycle regulation (Supplemental Fig. S1E), supporting the feasibility of using SpectralSeq to study cell molecular states.

Of the remaining 93 single cells in batch 1, 55 cells (59%) contained more than 25% of mitochondrial over total reads, an indicator of suboptimal cell viability (Fig. 1D; Wolf et al. 2018). The hyperspectral images were analyzed using principal component analysis (PCA) (Lever et al. 2017). When represented on the first two hyperspectral principal components, two groups of cells were observed (Supplemental Fig. S1F, separated by a straight line), confirming the suspicion that a subpopulation of cells might exhibit low viability due to the experimental protocol. In contrast, all 81 cells from batch 2 (collected with DAPI staining) (Supplemental Fig. S1G) passed quality controls and had <25% mitochondrial over total reads (Fig. 1E), confirming that DAPI-based viability filtering significantly improved transcriptomic quality. The number of cells obtained using different protocol versions (with or without DAPI imaging) and those included in this study's analyses are summarized in Supplemental Table S3.

Comparison of data from both batches with published bulk RNA-seq (Hatwik et al. 2023) confirmed that genes typically expressed by MCF-7 cells (Comşa et al. 2015) were also detected in cells processed with SpectralSeq, as expected (Supplemental Fig. S1H). The correlation between our data and the bulk RNA-seq replicates was ∼0.7, indicating overall agreement (Supplemental Fig. S1I). Hyperspectral PCA on both experimental batches combined again showed two groups of cells. MT-low cells (<25% mitochondrial over total reads) populated both groups (Fig. 1F, groups separated by a straight line). Within MT-low cells, the PCA group 1 was compared with the PCA group 2 for number of detected genes and percentage of ribosomal reads via two-tailed Kolmogorov–Smirnov (KS) tests (Supplemental Fig. S1K). MT-low cells in PCA group 1 showed a higher number of genes than MT-low cells in PCA group 2, whereas the percentage of ribosomal reads was similar. Differential expression analysis between the two groups of MT-low cells was also conducted (Fig. 1G). The top three upregulated genes in PCA group 1 versus 2 MT-low cells were *PPDPF*, involved in cell differentiation, Rho GDP Dissociation Inhibitor *ARHGDIA*, and *MTRNR2L12*, involved in negative regulation of the execution phase of apoptosis (Fig. 1G,H). Downregulated genes in the lower-left versus upper-right MT-low cells include kynureninase (*KYNU*), *RSU1*, which encodes a Ras Suppressor protein, and *CD47*, which encodes an adhesive protein mediating cell-to-cell interactions (Fig. 1G,H). Gene expression in MT-low cells only was highly concordant across batches (Supplemental Fig. S1L).

We also performed a simpler epifluorescence imaging experiment followed by picking and sequencing on three murine T cells to evaluate whether SpectralSeq is applicable to primary and nonadherent cells using microwells to help confinement (Supplemental Fig. S3A–C). The cells expressed T cell-specific genes (Supplemental Fig. S3B) and passed quality controls (Supplemental Fig. S3C), indicating that SpectralSeq does not impose major restrictions on the cell properties (e.g., size, origin, adhesion).

Overall, the combination of hyperspectral and transcriptomic information, with the added DAPI quality control step to remove apoptotic cells, enabled the detection of a population of cells that may be pre-apoptotic. This additional step led to an improved protocol with comparable throughput and flexibility.

### Characterization of cells with hyperspectral rings at the plasma membrane

Because SpectralSeq has subcellular optical resolution, we asked if the principal components of each cell's spectrum are related to spatial heterogeneity within each cell. The explained variance exhibited a gap after principal component 2 (PC2) (Supplemental Fig. S4A); therefore, we focused on the first two components as suggested by random matrix theory (Edelman and Rao 2005; Ergün 2009).

**Figure 2.**
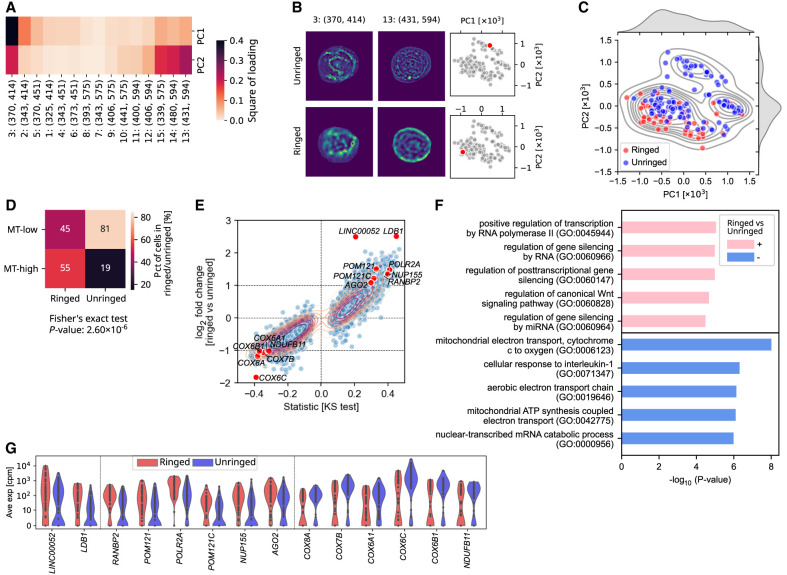
Optical cell heterogeneity can be used as a guide to query transcriptional heterogeneity. (*A*) The square of loading of every channel to PC1 and PC2 generated from PCA on average autofluorescent intensities under 15 channels of all cells. (*B*) Examples of segmented cells without (*upper* panel) and with (*lower* panel) bright rings in channel 13 (λ_ex_ = 431 nm, λ_em_ = 594 nm). The location of example cells in the PC1-PC2 map are shown in two *right* panels. (*C*) Scatter- and KDE plots of PC1 and PC2 of each cell. Red and blue face colors indicate cells with (ringed) and without (unringed) rings. (*D*) Heat map showing the percentage of ringed and unringed cells that are MT-low or MT-high. (*E*) Scatterplot of the log_2_ fold change between ringed and unringed cells (*y*-axis) and signed statistics from KS test (*x*-axis). Genes with median number in either subgroup as 0 were filtered out. (*F*) The top five Gene Ontology (GO) terms among genes upregulated or downregulated in ringed cells relative to unringed cells. Upregulated genes indicate genes with log_2_ fold change > 1 and −log_10_(*P*-value) > 2, and downregulated genes indicate genes with log_2_ fold change < −1 and −log_10_(*P*-value) > 2. Genes with median number in either subgroup as 0 were filtered out, and *P*-values were corrected by the Benjamini–Hochberg (nonnegative) method. (*G*) Violin plot showing the expression of some genes up- or downregulated in ringed cells.

PC1 and PC2 lent themselves to an intuitive optical interpretation: blue-emitting channels had larger squared loadings for PC1 and red-emitting channels for PC2 (Fig. 2A). Therefore, we examined their hyperspectral images further in channels 3 (λ_ex_ = 370 nm, λ_em_ = 414 nm) and 13 (λ_ex_ = 431 nm, λ_em_ = 594 nm), which had the largest squared loadings onto PC1 and PC2 (Fig. 2B). Some cells exhibited a ring at the plasma membrane in channel 13 (Fig. 2B, lower panel). A computational algorithm was built to classify cells with or without bright rings (see Methods; Supplemental Fig. S4B). It categorized 60 cells (34%) as ringed and 114 cells (65%) as unringed, which was confirmed visually. The majority of ringed cells (85%) were located in the PCA group 1 of the hyperspectral PCA (Fig. 2C). Moreover, the majority of ringed cells were MT-high, unlike unringed cells (Fisher's exact test, *P*-value 2 × 10^−6^) (Fig. 2D). Direct comparison between ringed and unringed cells revealed 272 differentially expressed genes (DEGs) with a large fold change and a strong statistical support (KS test) (Fig. 2E). Eighty-six percent (233 genes) were concordant between batches (Supplemental Fig. S4C). Further, correlation between expression of all DEGs and all optical channel intensities revealed that only channels with an emission wavelength of 575 nm showed bimodal distributions, indicating association with hyperspectral rings (Supplemental Fig. S4D). We performed a permutation analysis based on 1000 random shuffles of the “Ringed” and “Unringed” labels; results showed that 0 out of 1000 shuffles had a higher difference in mitochondrial over total reads between these two pseudo groups than the original result (Supplemental Fig. S4E), indicating that the observed difference is highly unlikely to have occurred by chance.

Enriched pathways among upregulated genes in ringed (+) and unringed (−) cells were computed via GSEApy (Fig. 2F; Fang et al. 2023). Three pathways enriched among genes upregulated in unringed cells were associated with mitochondrial electron transport, indicating a more active tricarboxylic acid (TCA) cycle and increased ATP production (Nolfi-Donegan et al. 2020). Genes within these pathways included *COX8A*, *COX7B*, *COX6A1*, *COX6C*, and *COX6B1* encoding cytochrome C oxidase subunits, along with *NDUFB11*, which encodes NADH:ubiquinone oxidoreductase subunit B11 (Fig. 2F,G). Genes upregulated in ringed cells had a less direct interpretation and were related in part to gene silencing (Fig. 2E–G). Ringed cells exhibited upregulated posttranscriptional gene regulation, resulting in a higher percentage of processed transcript RNA compared to unringed cells (Supplemental Table S4).

Overall, these data indicated that unringed cells might be metabolically more active, whereas ringed cells might have been in a less vital state at the time of sampling. More generally, SpectralSeq was effective at discovering subcellular optical features and generating data-driven hypotheses about their biological meaning.

### Cell size correlates with microtubule growth, and RNA splicing and degradation

**Figure 3.**
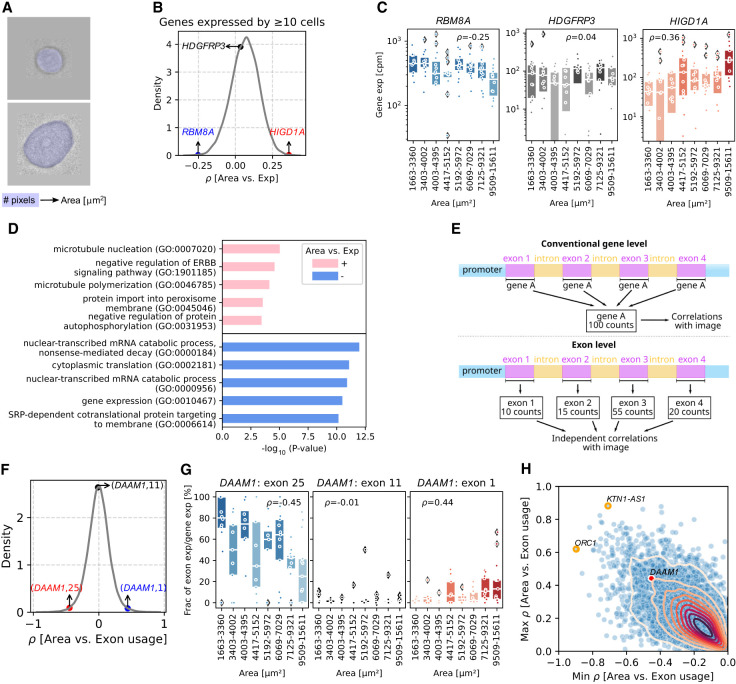
Correlating cell size with gene expression and relative exon usage. (*A*) Schematic plot of computing cell areas based on the number of pixels. (*B*) Kernel density estimate (KDE) plot of correlation coefficients between cell area and gene expression for genes expressed by ≥10 cells. Three genes with negative, ∼ zero, and positive correlation coefficients are labeled. (*C*) Box plots showing the gene expression (cpm) in every 15 cells ranking with cell areas from smallest to largest of genes in *B*. Each dot represents a cell, and box color is coded by the median expression in each bin. Box plots’ horizontal lines indicate the first, second (median), and third quartiles. Spearman's ρ-values: *RBM8A* = 0.006, *HDGFRP3* = 0.695, and *HIGD1A* = 5.52 × 10^−5^. (*D*) The top five Gene Ontology terms among 300 genes with highest positive or negative correlation coefficients with cell areas. Genes that are expressed with less than 10 cells are filtered out. (*E*) Schematic plot of transcriptomic analysis at gene level and exon level. (*F*) KDE plot of correlation coefficients between cell area and exon usage on exons from genes expressed by ≥10 cells. Three exons within one gene with negative, ∼ zero, and positive correlation coefficients are labeled. (*G*) Box plots showing the exon usage in every 15 cells ranking with cell areas from smallest to largest. Each dot represents a cell, and color is coded by the median usage in each bin. Box plots’ horizontal lines indicate the first, second (median), and third quartiles. Spearman's ρ-values: (*DAAM1*: exon 25) = 4.23 × 10^−5^, (*DAAM1*: exon 11) = 0.906, and (*DAAM1*: exon 1) = 6.05 × 10^−5^. (*H*) Scatterplot of the maximum (*y*-axis) and minimum (*x*-axis) correlation coefficients between exon usage and cell area within a gene. Genes that are expressed with less than 10 cells are filtered out. Gene *DAAM1* in *G* is labeled as red. Genes with smallest minimum correlation coefficient and largest maximum correlation coefficient are labeled with orange circles.

SpectralSeq can be used not only to investigate autofluorescence but also to associate optical features in the brightfield channel, such as cell size and morphology, with gene expression. MCF-7 cells picked from the same culture dish were heterogeneous in size. The largest cell (15.61 µm^2^) had an area nine times larger than the smallest one (1.66 µm^2^) (Fig. 3A; Supplemental Fig. S5A). Generally, cell size is determined by the relative rates of cell growth and division (Amodeo and Skotheim 2016) and can be manipulated genetically (Nurse 1975). Additional sources of heterogeneity beyond cell cycle might exist in the culture and be associated with detectable gene expression signatures.

To investigate the relationship between cell size and transcriptional profile, the expression of each gene was correlated with cell size. Because our previous data suggested that MT-high cells are apoptotic, these analyses used only MT-low cells. The distribution of correlation coefficients (Spearman's ρ) peaked around zero, indicating that most genes are evenly expressed across cell sizes (Fig. 3B; Supplemental Fig. S5B). Some genes were expressed at lower levels in larger cells (e.g., *RBM8A*) (Fig. 3C, left) and some genes at higher levels in larger cells (e.g., *HIGD1A*, previously implicated in pancreatic cancer cell growth [An et al. 2019]) (Fig. 3C). To understand the biology of genes associated with cell size, the most enriched pathways within genes with largest positive or negative correlation with cell size were computed via GSEApy (Fig. 3D; Fang et al. 2023). Pathways associated with positive correlation included microtubule nucleation and polymerization (Sulimenko et al. 2017), an indication that larger cells might be growing their cytoskeleton, perhaps in preparation for mitosis. Among the hits in this pathway was *TUBG2*, which encodes a protein required for microtubule nucleation at the centrosome (Supplemental Fig. S5C). Two of the pathways associated with negative correlation related to RNA degradation. Genes in these pathways included *RBM8A* (Fig. 3C, left), also known as *Y14*, and *EIF4A3*, both of which encode core members of the exon splicing junction complex (EJC) (Ballut et al. 2005; Chuang et al. 2015). *EIF4A3* encodes a DEAD-box protein that is bound to ATP, whereas the MAGOH-Y14 heterodimer inhibits EIF4A3 ATPase activity, destabilizing the association of the EJC with its RNA substrate.

To validate these results against external, independent data, we reanalyzed published scRNA-seq data on 977 MCF-7 cells (Chen et al. 2021), assigning cell size scores from 1 to 5 to phases M/G1, G1/S, S phase, G2, and G2/M based on the cell cycle. Cells with larger size scores expressed higher levels of microtubule organization genes and lower levels of cytoplasmic translation genes (Supplemental Fig. S5I), further buttressing our primary findings based on SpectralSeq.

Given that spliceosome factors were downregulated in larger cells, we suspected that cells of different size might show preferential usage of specific gene isoforms. To explore this hypothesis, HTSeq 2.0 was employed to subassign reads for individual genes into their exonic components (Putri et al. 2022). Within each gene, the fraction of reads assigned to each exon was computed in every cell (Fig. 3E). Fractional exon usage was then correlated with cell size, yielding a zero-centered distribution (Fig. 3F). To understand the meaning of this result, we considered *DAAM1*, a member of microfilament-related formins (Aspenström et al. 2006). Whereas exon 11 showed a similar fractional usage across all cell sizes (Fig. 3G, middle), exon 25 was preferentially used by smaller cells (Fig. 3G, left) and exon 1 by larger ones (Fig. 3G, right). The minimum and maximum correlation coefficients between exon usage and cell area were computed for each gene (Fig. 3H). The majority of genes were located close to the origin, indicating little exonic preference across cell sizes. A streak of genes was close to the diagonal, which is consistent with a maximum-parsimony model with only two preferential exons (one for small cells, one for large cells). Some genes were located off-origin and off-diagonal, suggesting more complex splicing preferences. Across the entire transcriptome, origin recognition gene *ORC1* showed the smallest minimum correlation, whereas long noncoding RNA *KTN1-AS1* showed the largest maximum correlation. Kolmogorov–Smirnov tests were also performed on exon usage between ringed and unringed cells (Supplemental Fig. S5D; Supplemental Table S5). No clear association between spliceosome and hyperspectral rings was found (Supplemental Table S6).

In addition to cell size, cell eccentricity (Supplemental Fig. S5E) was also computed and correlated with gene expression to exemplify the usage of more complex morphological features, resulting in a separate set of genes and pathways (Supplemental Fig. S5F–H). Overall, these findings indicate that the MCF-7 cell size heterogeneity is associated with preferential usage of biological pathways, single genes, and individual exons.

### SpectralSeq peers into the metabolic state of cells

**Figure 4.**
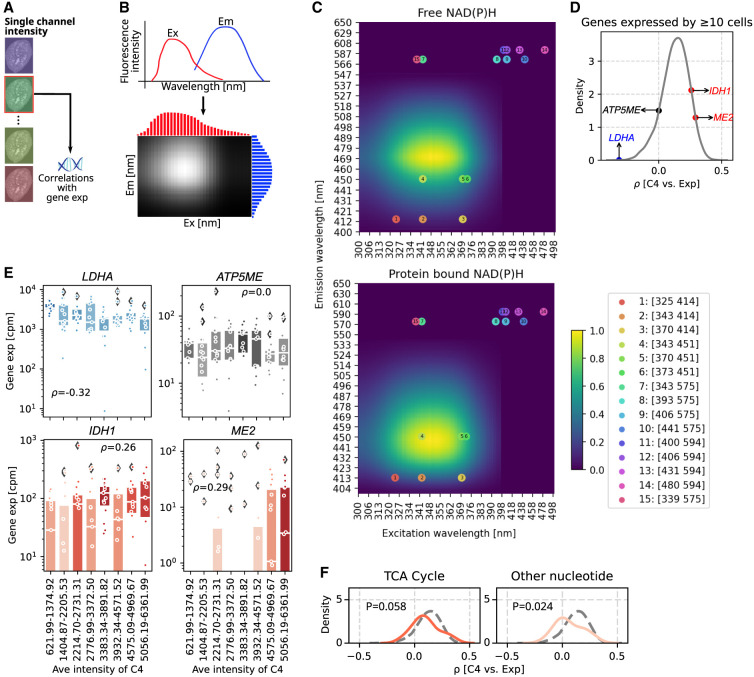
SpectralSeq generates hypotheses about cell metabolism. (*A*) Schematic of workflow to correlate a single channel intensity with gene expression. (*B*) Schematic plot of constructing spectra matrix using the excitation and emission spectra. (*C*) Spectra matrices of free (*top*) and protein-bound NAD(P)H (*bottom*). Dots represent the positions of the 15 hyperspectral channels according to the excitation and emission wavelengths, and numbers inside the dots correspond with the channel number in the legend box. (*D*) KDE plot of correlation coefficients between spectra intensities in channel 4 and gene expressions for genes expressed by ≥10 cells. Four genes with negative, ∼zero, and positive correlation coefficients are labeled. (*E*) Box plots showing the gene expression (cpm) in every 15 cells ranking with spectra intensities in channel 4 from smallest to largest of genes in *D*. Each dot represents a cell, and box color is coded by the median expression in each bin. Box plots’ horizontal lines indicate the first, second (median), and third quartiles. Spearman's ρ-values: *LDHA*=4.47 × 10^−4^, *ATP5ME* = 0.994, *IDH1* = 0.004, and *ME* = 0.001. (*F*) KDE plots showing the correlation coefficients between spectra intensity in channel 4 and gene expression of genes involved in two cellular metabolic pathways. Vertical gray dashed lines indicate correlation coefficient of −0.5, 0, and 0.5, and the horizontal gray dashed line indicates a KDE density of 5. The gray dashed curve presents the KDE distribution for all genes in the pathway (as in *D*). The color of the KDE distribution is coded by the correlation coefficient of the peak.

The hyperspectral information from each cell can also be used to study specific autofluorescent biomolecules. Nicotinamide adenine dinucleotide hydrogen (NADH) and its phosphate (NADPH), two enzyme cofactors and electron carriers playing ubiquitous roles in cell metabolism (Anderson et al. 2017; Xiao et al. 2018), are among the brightest autofluorescent biomolecules within human cells. A hyperspectral channel was therefore chosen to study NAD(P)H metabolism, aiming to correlate its intensity with gene expression from the same cell (Fig. 4A). Because autofluorescence is an optically incoherent process involving vibrational quantum states of the medium, the signal detected in the microscope camera is proportional to the direct product of excitation spectrum times emission spectrum at the chosen pair of wavelengths (Fig. 4B). Therefore, the hyperspectrum of free NAD(P)H was reconstructed from previously published spectra (Supplemental Fig. S6A; Schaefer et al. 2019) and intensity in channel 4 (λ_ex_ = 343 nm, λ_em_ = 451 nm) was chosen as a proxy for the spectral peak of this molecule (Fig. 4C, top). The correlation between channel 4 intensity with gene expression resulted in a distribution of correlation coefficients with a slight positive bias (Spearman's ρ) (Fig. 4D). Multiple genes related to the production of NADH had strong positive correlations with channel 4 intensity, including *IDH1* (ρ = 0.26) and *ME2* (ρ = 0.29) (Fig. 4E). *LDHA*, which encodes the enzyme LDHA that catalyzes NADH conversion into NAD+, had a strong negative correlation (ρ = −0.32) (Fig. 4E). These data indicate that hyperspectral imaging in channel 4, which is proximal to the NAD(P)H autofluorescence spectral peak, is consistent with a specific transcriptomic signature affecting NAD(P)H metabolism. We also performed a separate analysis on the ratio between channel 4 and channel 2 intensities, which is expected to contain information about the blue-shifted spectrum of protein bound NAD(P)H (Fig. 4C, bottom; Kasischke et al. 2011; Mahbub et al. 2017). That analysis indicated a more narrow variation in correlation coefficient and a partially overlapping set of genes (Supplemental Fig. S6B–D).

MCF-7 cells utilize glucose for about half of their oxidative metabolic energy needs, which requires TCA cycle enzymes to generate mitochondrial NADH (Quek et al. 2022). Therefore, we examined the distribution of correlation coefficient in the TCA cycle and nucleotide synthesis, which showed a slight tendency toward lower correlation coefficients (Fig. 4F; Supplemental Table S7). The robustness of these analyses were confirmed by permutation analysis on the intensities in channel 4 (Supplemental Fig. S6E,F). Taken together, these data suggest that SpectralSeq can be used to inform about the biology of genes and pathways directly and indirectly related to a chosen autofluorescent metabolite.

## Discussion

The integration of omics data with complementary measurements from the same cell has been limited by challenges in combining high-throughput single-cell assays into a single experimental approach (Mayr et al. 2019; Watson et al. 2022). Spatial transcriptomic technologies (Chen et al. 2015; Rodriques et al. 2019), although growing in popularity, currently suffer from either low spatial resolution or low RNA capture efficiency. Live-seq, which uses an atomic force microscope (Chen et al. 2022), can be time-consuming and requires a high level of expertise. Chip-based techniques are addressable and high-throughput but have limitations in imaging resolution (DeLaughter 2018; Kaya-Okur et al. 2019). SpectralSeq was designed to make integration of imaging and sequencing accessible to a broader audience of biomedical researchers. By collecting rich, label-free optical information on select cells of choice, SpectralSeq is ideally suited to study specific cellular phenotypes (e.g., particularly small/large cells), as well as cryptic phenotypes (e.g., ringed cells). Additionally, SpectralSeq can have better cost scalability (depending on the experimental design) because it enables the focus on specific cells matching a priori criteria. Notably, SpectralSeq separates the high-resolution imaging platform from the cell picker, which lifts the requirement to have a single, highly specialized setup. In our case, the two pieces of equipment were on adjacent floors of one building. It might be possible to transport the cells further if media is added after imaging. DAPI can be used to increase the fraction of high-quality cells captured (Supplemental Fig. S1G) to almost 100%, similar to fluorescence activated cell sorting (FACS) (Picelli et al. 2014). Future research is planned to extend the current scope to other cell lines, cells in suspension, mixed cell populations, and primary cells. Other potential extensions of SpectralSeq include chromatin accessibility (Chen et al. 2018; Zhang et al. 2022), whole-genome sequencing (Gonzalez-Pena et al. 2021) at the sequencing level, and analysis of subcellular organelles (Fu and Xie 2014; Husain et al. 2023) at imaging level. SpectralSeq could also be used on engineered cells that export samples of the transcriptome nondestructively (Horns et al. 2023), potentially achieving data sets similar to Live-seq with a simpler workflow.

SpectralSeq was used to explore transcriptomic differences across hyperspectral heterogeneity. Current applications of hyperspectral imaging remain at the stage of discerning different physiological states of cells and tissues based on their autofluorescent hyperspectrum—for example, the assessment of the viability of islets (Campbell et al. 2023c) and watermelon seeds (Yasmin et al. 2022). However, research aimed at probing the molecular distinctions underlying specific hyperspectral patterns is limited. In this study, we identified a subset of cells displaying multiband autofluorescence rings at the plasma membrane that also presented an altered transcriptome, with, potentially, upregulation of gene silencing and downregulation of cellular respiration. Further validations of this finding to identify healthier cells for downstream applications will be needed. This result provides a blueprint for future studies using SpectralSeq to reveal the transcriptomic changes underpinning spectral changes in specific subcellular compartments, such as the nucleus or mitochondria.

SpectralSeq can also be used to correlate gene expression with brightfield images. Regulation of cell morphology is complex (Tran et al. 2007; Shomroni et al. 2022); therefore, technical advances in this area would be important to better our understanding. Classic literature from yeast shows that genetically induced manipulation of the cell cycle affects cell size (Nurse 1975). However, studying the natural distribution of sizes, especially in multicellular organisms and with single cell resolution, is less common. A previous study reported several pathways involved in cell-size regulation by analyzing cells at different cell-cycle stages, but it did not focus on cell size directly or link it to transcriptomics at the single-cell level (Björklund et al. 2006). SpectralSeq can be a useful tool in this area given its applicability to many types of cells, both in culture and primary.

SpectralSeq is also applicable to investigate the relationship between alternative splicing and visual phenotypes such as cell morphology or autofluorescence. Unlike droplet-based methods (Klein et al. 2015; Zheng et al. 2017) which generate reads at one end of the transcript, SpectralSeq reads cover the entire gene body (Arzalluz-Luque and Conesa 2018), enabling read counting on a per-exon basis using HTSeq 2.0 (Putri et al. 2022) or similar tools. Although other computational approaches to single-cell isoform analysis such as SingleSplice (Welch et al. 2016), BRIE (Welch et al. 2016), and Expedition (Song et al. 2017) were not assessed in this study, they are expected to be compatible with SpectralSeq because the genomics data is in the same format as Smart-seq2 (Picelli et al. 2014). More tentatively, it might be possible to adapt the library preparation protocol to long-read sequencing technologies (e.g., Pacific Biosciences [PacBio], Oxford Nanopore Technologies [ONT]) (Arzalluz-Luque and Conesa 2018) to perform association analysis between full isoform abundance and hyperspectral features.

The transcriptomic proxies in living cells of autofluorescent biomolecules such as NAD(P)H can also be studied using SpectralSeq. NAD(P)H plays ubiquitous roles in cell metabolism (Anderson et al. 2017; Xiao et al. 2018). In addition, NADH binding sites are known to be altered by metabolic pathways related to carcinogenesis and differentiation, and enzymatic binding directly influences NADH cycling through energy production pathways (Banerjee and Bhatt 1989). For example, Bird et al. (2005) found that, in breast cancer cells, the ratio of free to protein-bound NADH is related to the NADH/NAD+ redox ratio (Bird et al. 2005). In this study, we peered into the metabolic states of MCF-7 cells by correlating transcriptomic expression with features extracted on hyperspectral channels designed for NAD(P)H. Whereas this study has focused on NAD(P)H, other biomolecules of importance for cell metabolism and behavior could be studied using the same concept, including flavins, collagen, and protoporphyrin IX (Campbell et al. 2022; Habibalahi et al. 2022). A limitation of this study is that optical features were not background-subtracted and calibrated into absolute metabolite concentrations. Future work is planned to address this point.

In conclusion, SpectralSeq was shown to be a robust approach to integrate hyperspectral imaging and transcriptomics at the single-cell level, illuminating the hidden relationship between a cell's appearance and its internal molecular state.

## Methods

### Cell culture

The human breast cancer cell line MCF-7 was purchased from American Type Culture Collection. MCF-7 cells were cultured in RPMI (Gibco) with 10% fetal bovine serum (FBS, Bovogen) and 4 mM L-glutamine (Thermo Fisher Scientific). The cells were seeded in a 35-mm coverslip-bottomed dish with an imprinted 500-µm cell location grid (ibidi) and cultured at 37°C in a humidified atmosphere with 5% CO_2_ for ∼18 h to allow adherence. To avoid overcrowding and minimize doublet or multiplet capture from strong cell-cell adhesion, cell density was kept at ∼5000 cells per dish (Supplemental Fig. S1G).

### Hyperspectral imaging

MCF-7 cells were washed twice with PBS, then placed into equilibrated Hank's Balanced Salt Solution (HBSS) (Thermo Fisher Scientific) and maintained at 37°C during imaging. Hyperspectral imaging was carried out with a customized fluorescence microscope (Olympus IX83) with a 40× oil objective (NA 1.35, Olympus), an iXon Ultra 888 EMCCD (model: hnu1024, Andor) and operated below −30°C to reduce noise. Custom-designed LED illumination and filter cubes enabled autofluorescence imaging in 15 channels (excitation values are ±5 nm and emissions are ±20 nm) (Supplemental Table S1).

### Single-cell capture

#### MCF-7 cells

After hyperspectral imaging, MCF-7 cells in the gridded dish were washed twice with PBS. To reduce adherence of MCF-7 cells without causing excessive motility, cells were treated with 10% TrypLE Express Enzyme (no phenol red, Thermo Fisher Scientific) for 30 min at room temperature before capture. Preliminary tests indicated that specific concentration and treatment time vary by cell line. Cells were identified on the CellCelector platform (Sartorius AG) and picked by a 30-µm glass capillary under manual picking mode. The captured cells were dispensed with 0.2 µL DNase/RNase-Free Water (Thermo Fisher Scientific) into a 384-well Frame-Star plate (4titude Ltd) with 1 µL lysis buffer. The lysis plate was cooled down to 4°C at the rack tray during cell capture and was stored at −80°C for subsequent procedures. In batch 2, DAPI (1:1000) (Thermo Fisher Scientific) was added into PBS supplemented with 10% TrypLE Express Enzyme 10 min before capture. Both DAPI and brightfield channels of CellCelector were used to identify cells. Inside the imaged grids, non-DAPI fluorescent cells were collected using the same procedure.

#### Murine primary T cells

Lymph nodes from OT-1-Thy1.1 mice (cross of OT-1 and Thy1.1 lines from Jackson Laboratories) were dissociated into single-cell suspension. CD8^+^ T cells were isolated (EasySep Mouse CD8^+^ T Cell Isolation kit) and labeled by CellTrace carboxyfluorescein succinimidyl ester (CFSE) (Thermo Fisher Scientific). T cells were seeded in nanowell slides (well size 40 µm, depth 40 µm, 50,000 nanowells/well) (Sartorius AGy). T cells were picked using a 30-µm glass capillary in automatic mode and dispensed with 0.1 µL DNase/RNase-free water into a 384-well Frame-Star plate containing 1.1 µL lysis buffer. The plate was cooled to 4°C during capture and stored at −80°C.

### Single-cell transcriptomic library preparation

Library preparation followed the Smart-seq2 protocol (Picelli et al. 2014). Cells were picked into 384-well lysis plates followed by reverse transcription, template switching, and PCR (26 cycles for MCF-7 cells and 23 cycles for T cells) to generate and amplify cDNA. A MANTIS automated liquid handler (Tecan) was used during the process. cDNA was normalized to 0.1–1 ng/µL, as qualified by a Quant-iT PicoGreen dsDNA Assay kit (Thermo Fisher Scientific) via the automated liquid handling robot Mosquito (SPT Labtech). A Nextera XT kit (Illumina) with 14 amplification cycles was used for tagmenting MCF-7 cells, whereas a TruePrep DNA Library Prep kit V2 (Vazyme #TD503) with 15 amplification cycles was used for immune cells. Purification was done by Agencourt Ampure XP magnetic beads (0.7× ratio, two rounds) (Beckman Coulter). Libraries were quantified using Bioanalyzer 2100 (Agilent Technologies).

### High-throughput sequencing

All libraries were sequenced at Ramaciotti Centre for Genomics (UNSW). The batch 1 library was sequenced on NextSeq 500 (Illumina) with 75-base paired-end reads. The batch 2 library was sequenced on NextSeq 1000 (Illumina) with 150-base paired-end reads. The pilot library was sequenced on MiSeq (Illumina) with 75-base paired-end reads. The T cell library was sequenced on NextSeq 500 (Illumina) with 2 × 75 paired-end reads. Sequencing coverage was ∼500,000–5,000,000 read pairs per cell.

### scRNA-seq data analysis

The following open source software was used for this study: numpy (Harris et al. 2020), pandas (McKinney 2010), Matplotlib (Hunter 2007), seaborn (Waskom 2021), SciPy (Virtanen et al. 2020), and SCANPY (Wolf et al. 2018).

### Preprocessing of scRNA-seq data

The sequencing reads were demultiplexed using bcl2fastq 2.20 (Illumina), mapped, and counted to human genome reference (GRCh38) for MCF-7 cells using STAR aligner (Dobin et al. 2013). HTSeq 2.0 (Putri et al. 2022) was used to map the sequencing reads of MCF-7 cells into exon level. Quality of data sets were evaluated based on the number of genes expressed in the count matrix, the total counts per cell, and the percentage of counts in mitochondrial genes. Genes that were expressed in less than 10 cells were excluded. Data sets were normalized by the total reads, multiplied by 1,000,000 (cpm). GSEAPY (Fang et al. 2023) was used for pathway analysis.

### Image analysis and cell segmentation

Hyperspectral figures were normalized by the most frequent fluorescence intensity in each channel, regarded as background intensity, and then scaled by 30, a value close to the background intensity. Pseudo RGB figures were created using channels 1, 6, and 13 to distinguish cells from the background. Rough single-cell positions were identified using Pixel Classification function in Ilastik (Supplemental Fig. S1D, left; Berg et al. 2019) on pseudo RGB figures. A custom semiautomatic tkinter-based GUI was developed to define cell boundaries (Supplemental Fig. S1D, right).

### Validation of cells inside PCA group 1

To distinguish between the two cell subpopulations identified by PC1 and PC2 from PCA analysis of the 15 average optical features of all cells, the boundary of PCA group 1 was determined as intensity value of 0.18 from kernel density estimation (KDE) analysis using SciPy (Supplemental Fig. S1J; Virtanen et al. 2020).

### Validation of cells with bright border rings

To determine if a cell exhibits a bright border ring at the plasma membrane, we segmented the border ring and the adjacent inner ring, each with a thickness of 10 pixels (Supplemental Fig. S4B). Subsequently, log_2_ fold changes between the average fluorescence intensity of the border and inner rings in channel 13 (λ_ex_ = 431 nm, λ_em_ = 594 nm) were computed. Cells with log_2_ fold changes greater than 0.4 were categorized as “ringed cells,” and those with log_2_ fold changes less than 0.4 were categorized as “unringed cells.”

### Hyperspectral imaging feature extraction

#### Machine learning features

Principal component analysis (Lever et al. 2017) was conducted on 15 average optical features of all cells using SciPy (Virtanen et al. 2020). Computational features with high cumulative explained variances were selected for further analysis.

#### Morphological features

Cell area and cell eccentricity that describe cell morphology were designed. Cell area was computed as the pixel counts of the segmented cell (Fig. 3A), whereas eccentricity that reflects cell shape was calculated as *y*/*x*–1 (*y*: cell length, *x*: width) (Supplemental Fig. S5E).

#### Optical features

Images of segmented cells were first processed by Gaussian blur in scikit-image (van der Walt et al. 2014) to reduce noise. Average fluorescence intensities of each cell in each channel were computed as optical features (Fig. 4A). Intensity ratios were defined as the ratios between average fluorescence intensities of two different channels (Supplemental Fig. S6B).

### Construction of spectra matrices of autofluorescent biomolecules

In order to get the absorbance of molecules in multiple channels, the excitation (Ex) and emission (Em) spectra of molecules were downloaded from published papers (Schaefer et al. 2019). Spectra matrices were constructed based on both Ex and Em spectra (Fig. 4B). In particular, the fluorescent intensities at different Ex and Em wavelengths were calculated according to Equation 1:

(1) $$I_{i}=(I_{\mathrm{Exi}}/\;I_{\mathrm{Ex0}})\times (I_{\mathrm{Emi}}/\;I_{\mathrm{Em0}})$$

where Ex0 and Em0 represent the Ex and Em with the highest fluorescent intensity in the spectrum, respectively, Exi and Emi are the Ex and Em under the *i*th condition, *I*_i_: normalized fluorescence intensity at Exi and Emi, *I*_Exi_ and *I*_Ex0_ represent the fluorescent intensity of Exi and Ex0 in the Ex spectrum, and *I*_Emi_ and *I*_Em0_ mean the fluorescent intensity of Emi and Em0 in the Em spectrum.

### Correlation analyses between hyperspectral, morphological, and optical features, and gene expression/exon usage

To keep enough genes for further study, genes expressed by ≥10 cells were selected for correlation analyses. The expression of each gene was correlated with distinct features by Spearman's correlation in MT-low cells only (117 cells in total). The distribution of correlation coefficients became closer to a normal distribution with an increase in the number of cells expressing genes because of reduced noise from ties in the cell ranks. Box plots were created to display representative genes from correlation analyses with gene expression (cpm) in cells, ranked from smallest to largest based on various features. The first seven boxes each comprised 15 cells, whereas the last box contained 12 cells.

### Pathway enrichment analysis on external, published scRNA-seq data

To validate these results against external, independent data, a published scRNA-seq data set of 977 MCF-7 cells (Chen et al. 2021) was used. Cell size scores from 1 to 5 were assigned to phases M/G1, G1/S, S phase, G2, and G2/M, separately based on the cell cycle. The top 300 genes with highest positive or negative correlation coefficients between cell size scores and the expression were used for pathway enrichment analysis.

## Data access

All raw and processed sequencing data generated in this study have been submitted to the NCBI Gene Expression Omnibus (GEO; https://www.ncbi.nlm.nih.gov/geo/) under accession number GSE254034. Python 3 and Jupyter notebooks used for the analysis are available at GitHub (https://github.com/echosun77/MCF7_hyperspectral_imaging_sequencing) and as Supplemental Code.

## Supplemental Material

Supplement 1

Supplement 2

Supplement 3

Supplement 4
