# Supplementary material for: Integration of hyperspectral imaging and transcriptomics from individual cells with SpectralSeq
Source: Genome Res. 2025 Aug;35(8):1809–20. doi: 10.1101/gr.280014.124 (PMC12315715; doi:10.1101/gr.280014.124)
Supplement: Supplement 2 [file Supplemental_File_S1.pdf]

Supplemental File S1. The before-and-after capture images from Cellcelector of doublets in Batch 1.

Cell	Before	After	Doublets
F3_S4	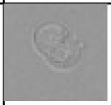	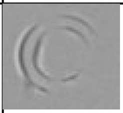	Yes
G3_S5	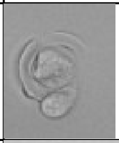	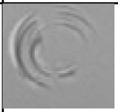	Yes
G4_S18	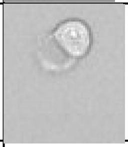	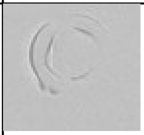	Yes
I4_S20	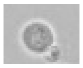	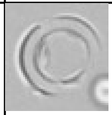	Yes
O4_S26	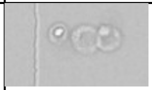	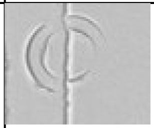	Yes
H5_S32	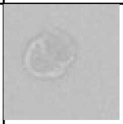	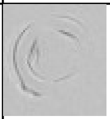	Yes
D6_S41	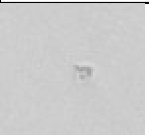	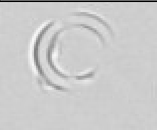	Yes
L6_S49	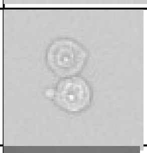	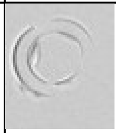	Yes
O7_S65	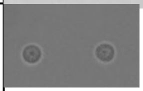	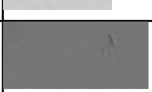	Yes
L8_S75	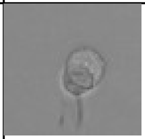	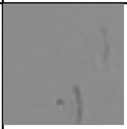	Yes
M8_S76	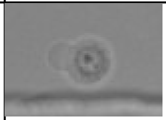	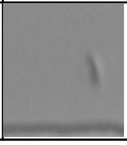	Yes

N8_S77	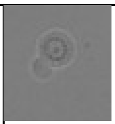	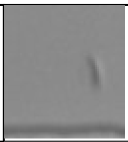	Yes
K9_S87	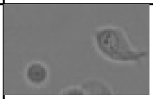	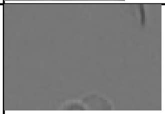	Yes
L9_S88	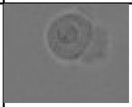	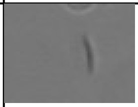	Yes
M9_S89	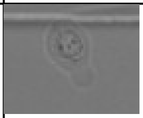	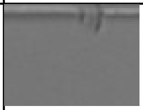	Yes
N9_S90	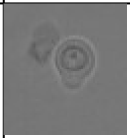	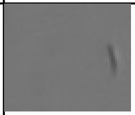	Yes
O9_S91	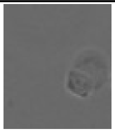	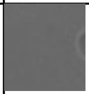	Yes
E10_S94	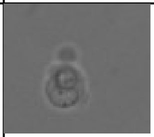	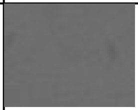	Yes
F10_S95	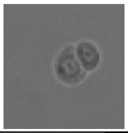	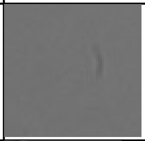	Yes
J10_S99	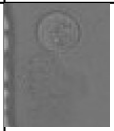	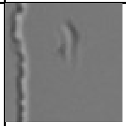	Yes
M10_S102	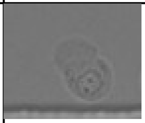	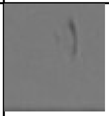	Yes
I11_S111	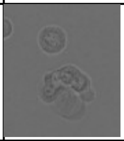	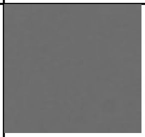	Yes
M11_S115	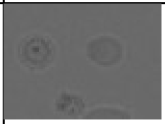	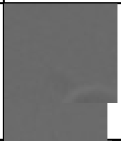	Yes

D12_S119	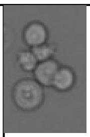	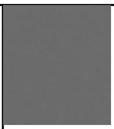	Yes
E12_S120	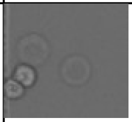	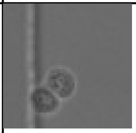	Yes
I12_S124	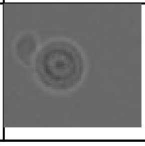	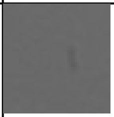	Yes
