# Supplementary material for: Integration of hyperspectral imaging and transcriptomics from individual cells with SpectralSeq
Source: Genome Res. 2025 Aug;35(8):1809–20. doi: 10.1101/gr.280014.124 (PMC12315715; doi:10.1101/gr.280014.124)
Supplement: Supplement 3 [file Supplemental_Figures.pdf]

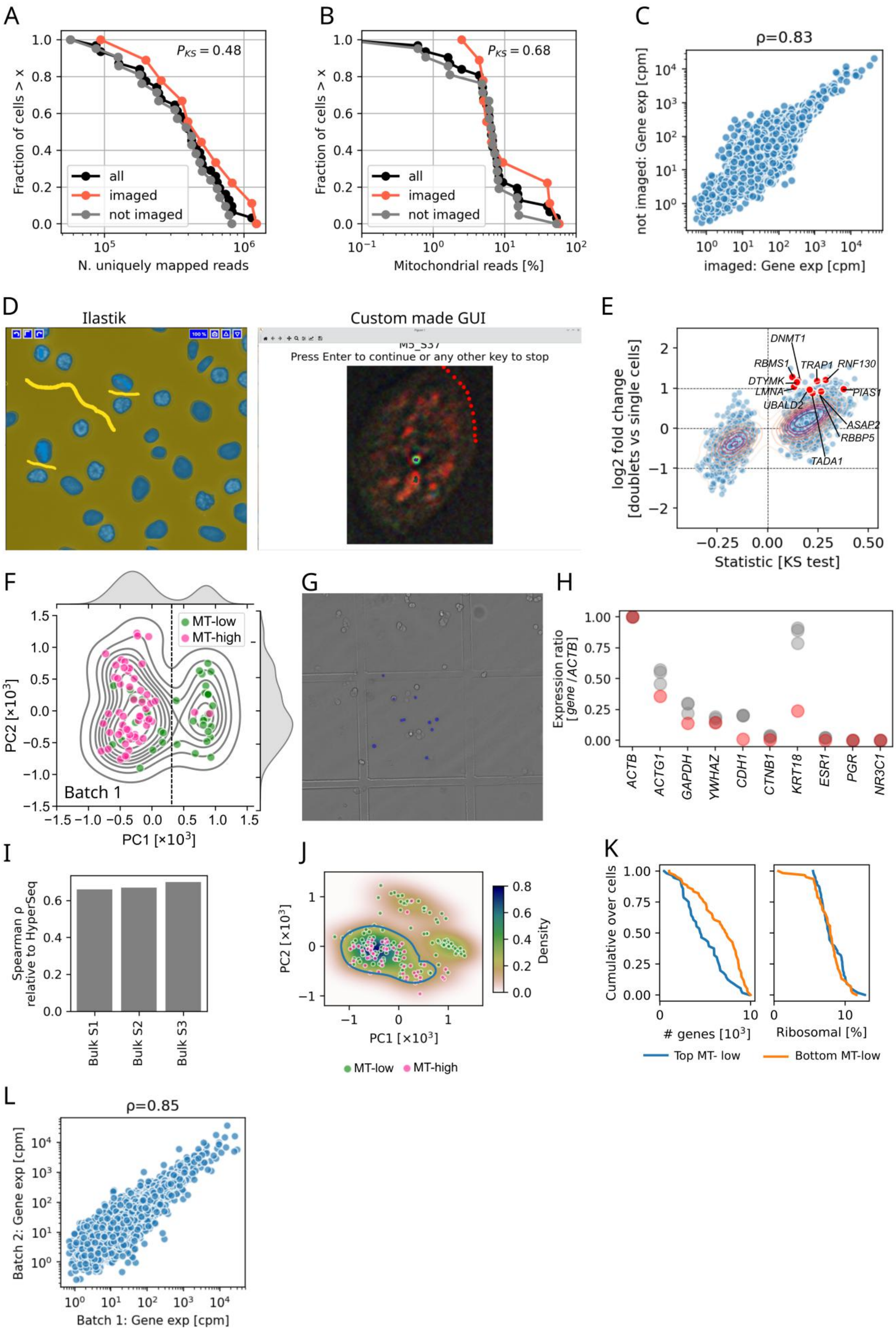
A
Fraction of cells > x
$P_{KS} = 0.48$
all
imaged
not imaged
N. uniquely mapped reads
B
$P_{KS} = 0.68$
all
imaged
not imaged
Mitochondrial reads [%]
C
ρ=0.83
not imaged: Gene exp [cpm]
imaged: Gene exp [cpm]
D
Ilastik
Custom made GUI
Press Enter to continue or any other key to stop
E
log2 fold change [doublets vs single cells]
DNMT1
RBMS1
TRAP1
RNF130
DTYMK
LMNA
UBALD2
PIAS1
ASAP2
RBBP5
TADA1
Statistic [KS test]
F
MT-low
MT-high
Batch 1
PC2 [×10³]
PC1 [×10³]
G
H
Expression ratio [gene / ACTB]
ACTB
ACTG1
GAPDH
YWHAZ
CDH1
CTNB1
KRT18
ESR1
PGR
NR3C1
I
Spearman ρ relative to HyperSeq
Bulk S1
Bulk S2
Bulk S3
J
PC2 [×10³]
PC1 [×10³]
Density
MT-low
MT-high
K
Cumulative over cells
genes [10³]
Ribosomal [%]
Top MT- low
Bottom MT-low
L
ρ=0.85
Batch 2: Gene exp [cpm]
Batch 1: Gene exp [cpm]

Supplemental Figure S1. (**A**, **B**) RNA quality control by comparing imaged cells and non-imaged cells in the pilot experiment. (**C**) Correlation of gene expression between imaged and unimaged cells in the pilot experiment. Each dot corresponds to one human gene. The correlation test (against a null hypothesis that the experiments are uncorrelated) was highly significant (p-value below machine precision). Only genes expressed by more than 3 cells in each sub group are shown to reduce visual crowding. (**D**) Schematic plots of Ilastik (left) and the custom made GUI (right) for cell segmentation. (**E**) Scatter plot of the $\log_2$ fold change between doublets and single cells in batch 1 (y-axis) and signed statistics from KS test (x-axis). Genes with median number in either subgroup as 0 were filtered out. (**F**) Hyperspectral principal component analysis (PCA) of experimental batch 1 highlighting two groups of cells, divided by a straight line. (**G**) Image of MCF-7 cells on the gridded plate after imaging. This image was constructed by both brightfield and DAPI fluorescent images. Cells masked with blue colour indicate DAPI-positive cells under fluorescent microscope. The DAPI mask was defined by the threshold of DAPI fluorescent intensity. (**H**) Comparison in gene expression between MCF-7 cells processed with HyperSeq (both batches) and an external bulk RNA-seq experiment on MCF-7 cells (Hatwik et al. 2023). Only control samples from the external experiment are shown. Each dot is a replicate. Expression levels are shown as ratios to *ACTB* for housekeeping genes (*ACTG1*, *GAPDH*, and *YWHAZ*) and other genes typically expressed by MCF-7 cells (*CDH1*, *CTNNB1*, *KRT18*, *ESR1*, *PGR*, and *NR3C1*. (**I**) Spearman correlation coefficients between each of bulk RNA-seq samples and HyperSeq (both bacthes) in (H). Genes expressed by all three bulk RNA-seq control samples and by more than 10 cells in HyperSeq are included. (**J**) Scatter plot of PC1 and PC2 of each cell. The contour of PCA group 1 was drawn as the density from KDE analysis on PC1 and PC2 as 0.18. (**K**) Distribution of the number of genes and percentage of MT-low cells inside or outside of the lower-left subgroup. (left: p-value below machine precision, right: p-value equels 0.21). (**L**) Comparison of biological replicates (batches 1 and 2) for MT-low cells. Correlation was computed by Spearman's ρ, and p-value is below machine precision. Each dot is a gene. Only genes expressed by more than 10 cells in each batch are shown to reduce visual crowding.

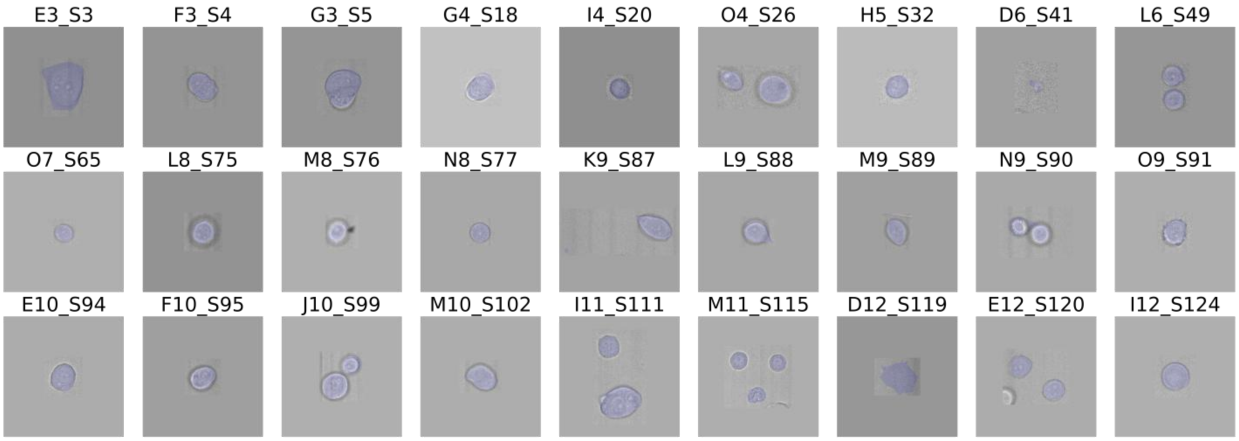

Supplemental Figure S2. Figure showing areas of doublets in batch 1. Cells from optical images were plotted with blue masks to be distinguished from the background.

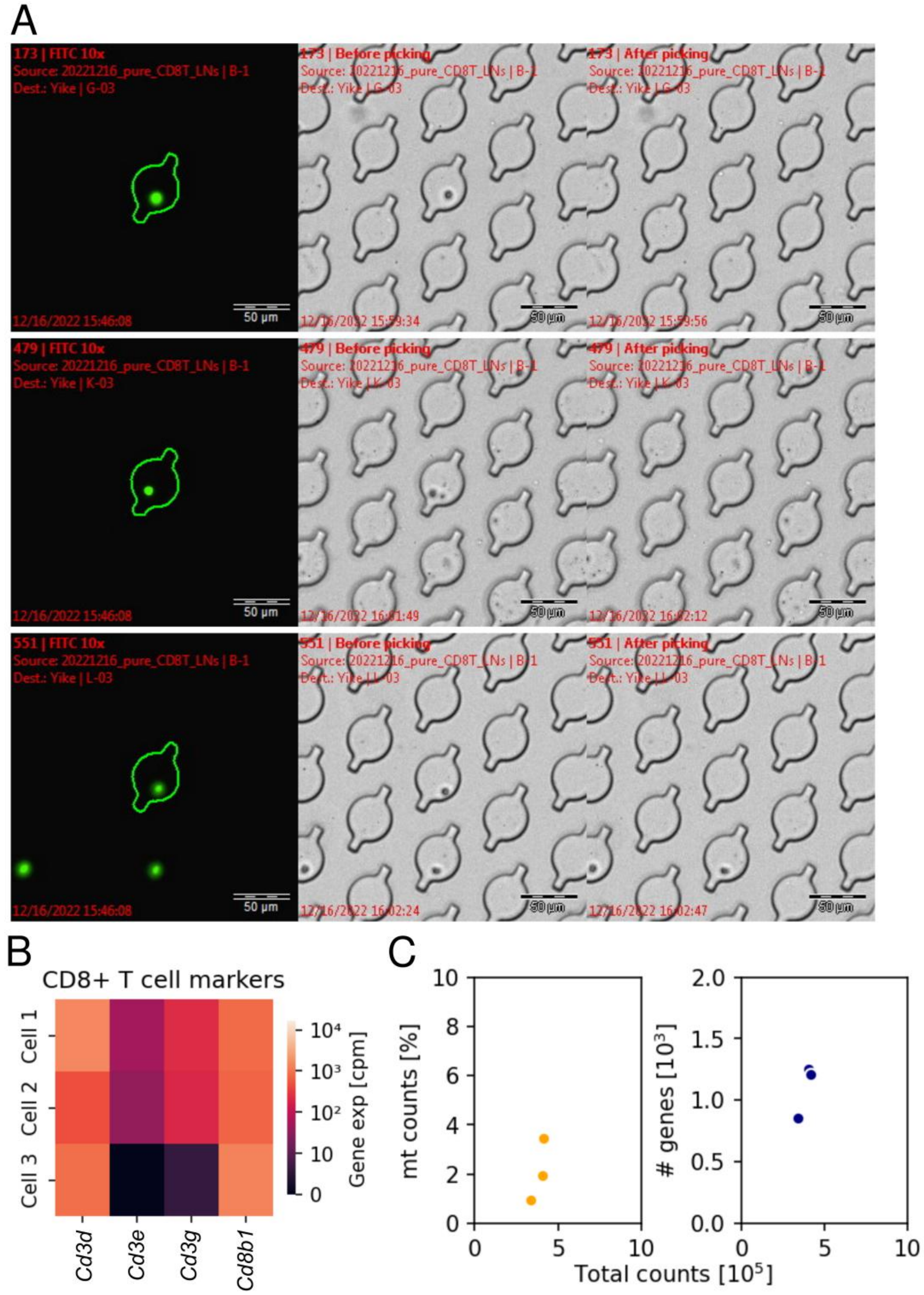

Supplemental Figure S3. (**A**) Fluorescent (left) and brightfield (middle) images before and brightfield image after (right) three primary murine T cells. Cells were seeded in nanowells and labelled by carboxyfluorescein succinimidyl ester (CFSE). See Methods for details. The green outline indicates the border of the chosen nanowell. (**B**) Expression of T cells genes in the three cells shown in (A). Cells 1, 2, 3 refer to top, middle, and bottom, respectively. (**C**) Total reads per cell versus percentage of mitochondrial over total reads for cells in (A).

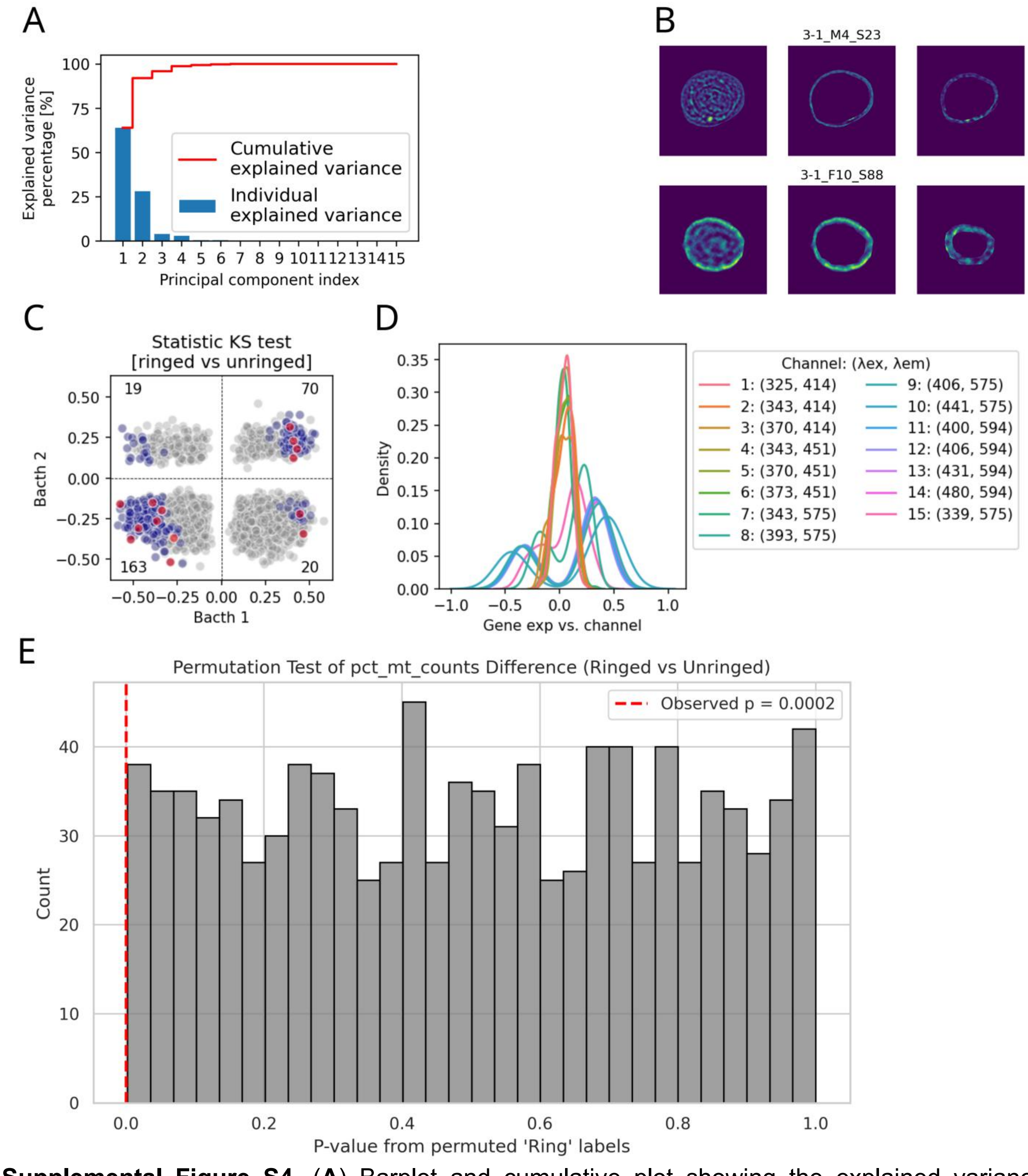

Supplemental Figure S4. (**A**) Barplot and cumulative plot showing the explained variance percentage (%) of each principal component (PC) from PCA on 15 channels. (**B**) Schematic plot of the border and inner rings segmented by distance of 10 pixels of cells without (the cell in **Fig. 2B**, upper panel) and with (the cell in **Fig. 2B**, lower panel) bright rings in channel 13. (**C**) Comparison between batches in terms of differential expression between ringed and unringed cells. For each axis (x and y), the Kolmogorov-Smirnov statistic of each gene (dot) between ringed and unringed cells within the same batch is shown. Blue + red dots indicate significant genes in the main analysis, i.e. the batches were merged (genes used for pathway analysis in **Fig. 2F**). (**D**) Distributions of correlation between expression of 272 select genes associated

with rings (genes used for pathway analysis in **Fig. 2F**) and all optical channel intensities. Correlation was computed by Spearman's ρ. (**E**) Histogram of P-values from 1,000 random shuffles of the "Ringed" and "Unringed" labels (defined in **Fig. 2C**) for differences in mitochondrial over total reads. The red dashed line marks the observed P-value from the original grouping.

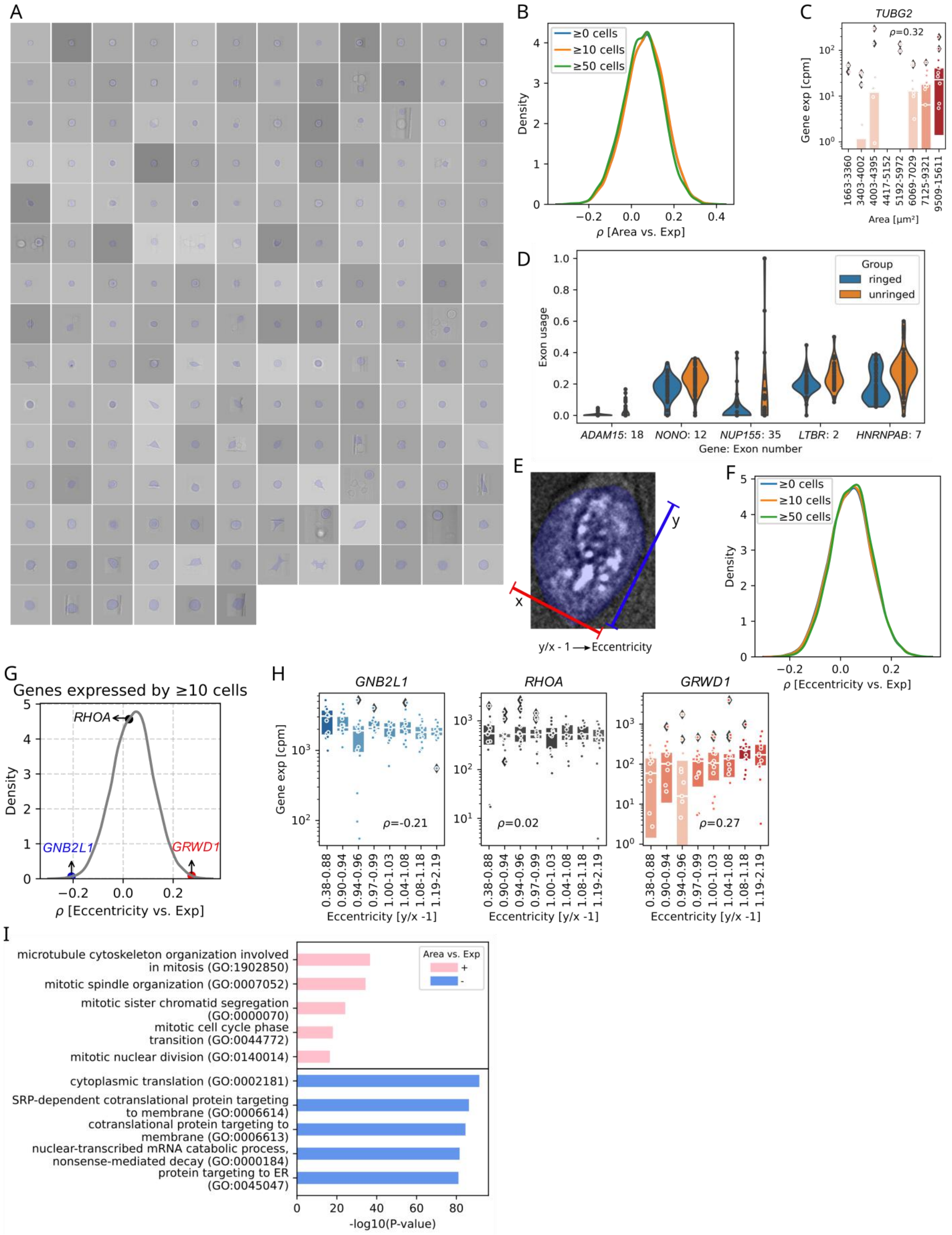

Supplemental Figure S5. (**A**) Figure showing areas of all cells based on cell area from lowest to largest. Cells from optical images were plotted with blue masks to be distinguished from the background. (**B**) KDE plot of correlation coefficients between cell area and gene expression on

genes expressed by ≥ 0, 10, and 50 cells. (**C**) Box plot showing the expression (cpm) of TUBG2 in every 15 cells ranking with cell areas from smallest to largest of a gene exclusively expressed in larger cells. Each dot represents a cell, and box colour is coded by the median expression in each bin. Box plots' horizontal lines indicate the first, second (median) and third quartiles. (**D**) Exons with detected differential usage between ringed and unringed cells. Each pair of violins refers to one exon within one human gene. Both batches were analysed jointly. (**E**) Schematic plot of computing cell eccentricities. (**F**) KDE plot of correlation coefficients between cell eccentricity and gene expression on genes expressed by ≥ 0, 10, and 50 cells. (**G**) KDE plot of correlation coefficients between cell eccentricity and gene expression for genes expressed by ≥ 10 cells. Three genes with negative, ~zero, and positive correlation coefficients are labelled. (**H**) Box plots showing the gene expression (cpm) in every 15 cells ranking with cell eccentricities from smallest to largest of genes in (G). Each dot represents a cell, and box colour is coded by the median expression in each bin. Box plots' horizontal lines indicate the first, second (median) and third quartiles. Spearman p-values: *GNB2L1*=0.023, *RHOA*=0.802, and *GRWD1*=0.003. (**I**) The top 5 gene ontology (GO) terms among 300 genes with highest positive or negative correlation coefficients with cell size score, defined based on on their cell cycle phase in the external dataset GSE144320 as described in the Methods. Genes that are expressed with less than 10 cells are filtered out.

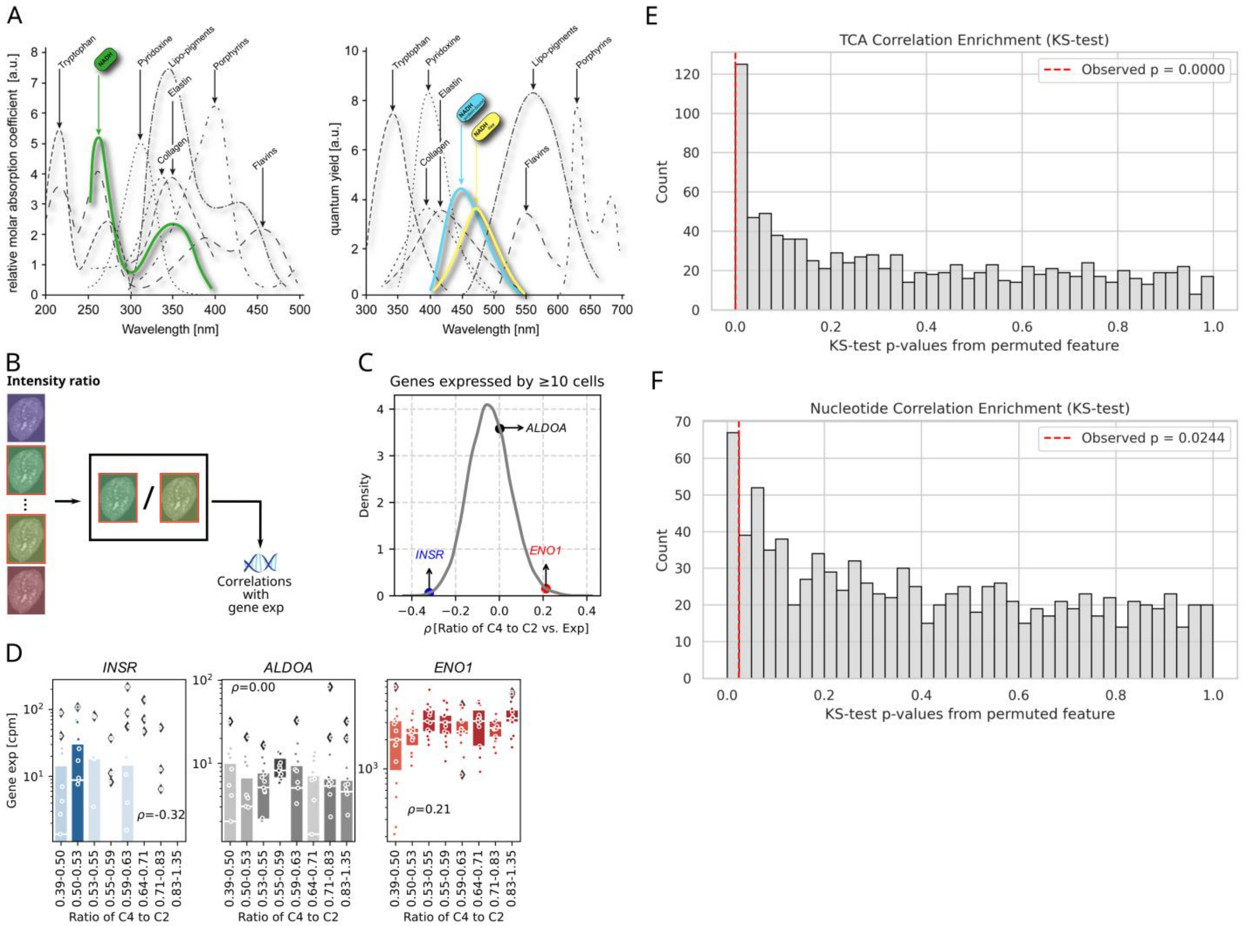

Supplemental Figure S6. (**A**) The absorbance (left) and respective emission (right) spectra of typical autofluorescent molecules with NADH highlighted. The height of the peaks indicates the relative molar absorption coefficient and the quantum yield, respectively. Image from Schaefer et al. 2019. (**B**) Schematic plots of correlations between the intensity ratio and gene expression. (**C**) KDE plot of correlation coefficients between the intensity ratio of channel 4 to channel 2 and gene expressions for genes expressed by ≥ 10 cells. Three genes with negative, ~ zero, and positive correlation coefficients are labelled. (**D**) Box plots showing the gene expression (cpm) in every 15 cells ranking with the ratio of intensity ratios of channel 4 to channel 2 from smallest to largest of genes in (C). Each dot represents a cell, and box colour is coded by the median expression in each bin. Box plots' horizontal lines indicate the first, second (median) and third quartiles. Spearman p-values: *INSR*=3.57×10^{-4}, *ALDOA*=0.970, and *ENO1*=0.019. (**E**-**F**) The histogram displays the distribution of Kolmogorov–Smirnov (KS) test P-values obtained from 1,000 random shuffles of the channel 4 (λ_{ex}=343nm, λ_{em}=451nm) values across cells. KS tests compare correlation coefficients between genes in the TCA cycle (E) or other nucleotide metabolism (F) and all genes. The red dashed line indicates the observed P-value from the original grouping.
