# Supplementary figures and images for: Integration of hyperspectral imaging and transcriptomics from individual cells with SpectralSeq
Source: Genome Res. 2025 Aug;35(8):1809–20. doi: 10.1101/gr.280014.124 (PMC12315715; doi:10.1101/gr.280014.124)

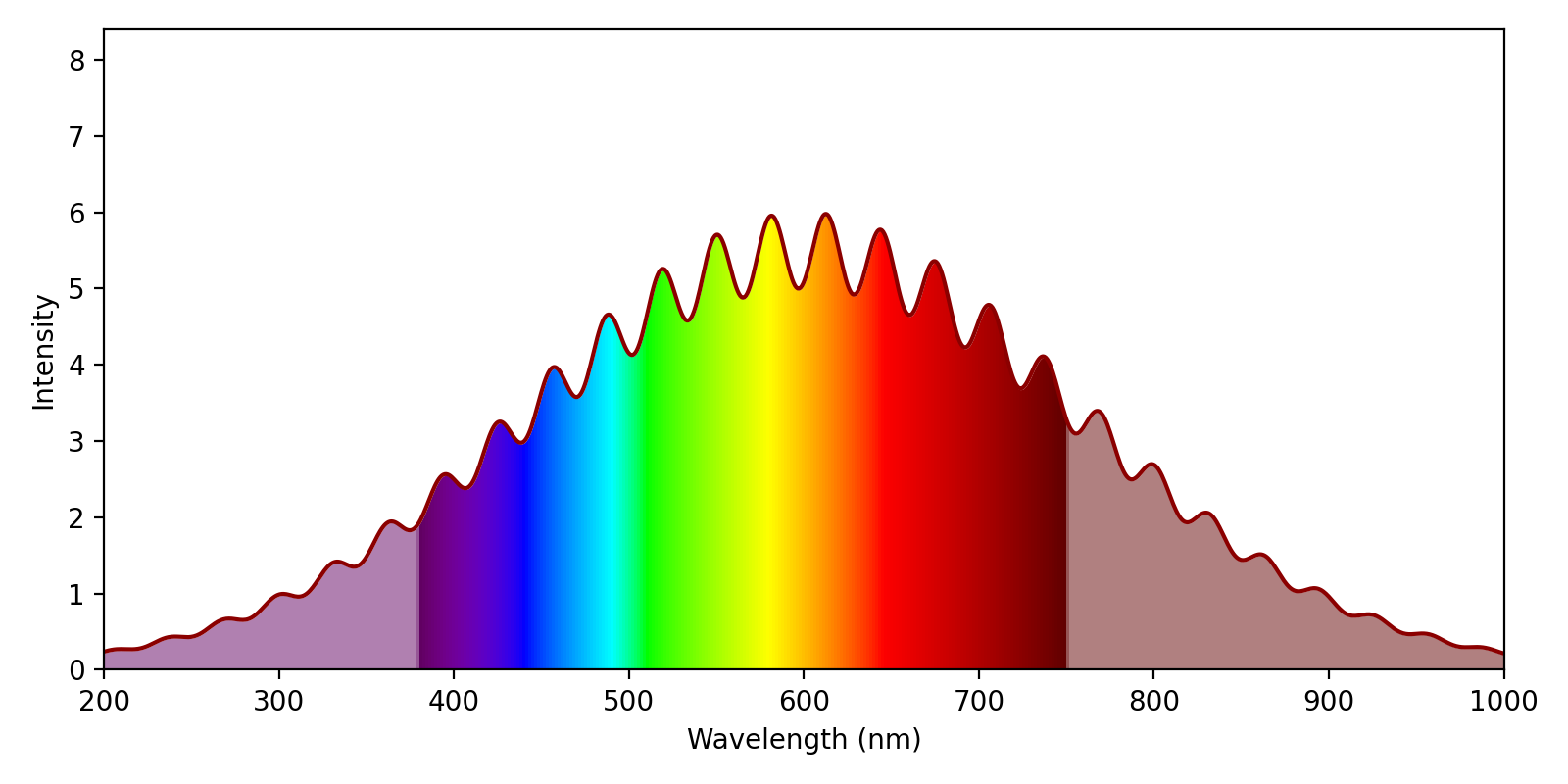

Supplement: Supplement 4 [file Supplemental_code.zip › Supplemental_code/figures/figure1/WavelengthColors.png]
